# Airborne particulate matter, population mobility and COVID-19: a multi-city study in China

**DOI:** 10.1186/s12889-020-09669-3

**Published:** 2020-10-21

**Authors:** Bo Wang, Jiangtao Liu, Yanlin Li, Shihua Fu, Xiaocheng Xu, Lanyu Li, Ji Zhou, Xingrong Liu, Xiaotao He, Jun Yan, Yanjun Shi, Jingping Niu, Yong Yang, Yiyao Li, Bin Luo, Kai Zhang

**Affiliations:** 1 Institute of Occupational Health and Environmental Health, School of Public Health, Lanzhou University, Lanzhou, Gansu 730000 People’s Republic of China; 2 Shanghai Key Laboratory of Meteorology and Health, Shanghai Meteorological Bureau, Shanghai, 200030 People’s Republic of China; 3 Department of General Surgery, the First Hospital of Lanzhou University, Lanzhou, Gansu 730000 People’s Republic of China; 4 Institute of Epidemiology and Statistics, School of Public Health, Lanzhou University, Lanzhou, Gansu 730000 People’s Republic of China; 5 Division of Social and Behavioral Sciences, School of Public Health, University of Memphis, Memphis, TN 38152 USA; 6 Department of Biostatistics and Data Science, School of Public Health, The University of Texas Health Science Center at Houston, Houston, TX 77030 USA; 7 Shanghai Typhoon Institute, China Meteorological Administration, Shanghai, 200030 China; 8 Department of Epidemiology, Human Genetics and Environmental Sciences, School of Public Health, The University of Texas Health Science Center at Houston, Houston, TX 77030 USA; 9 Southwest Center for Occupational and Environmental Health, School of Public Health, The University of Texas Health Science Center at Houston, Houston, TX 77030 USA

**Keywords:** COVID-19, Particulate matter, Population mobility, Generalized additive models

## Abstract

**Background:**

Coronavirus disease 2019 (COVID-19) is an emerging infectious disease, which has caused numerous deaths and health problems worldwide. This study aims to examine the effects of airborne particulate matter (PM) pollution and population mobility on COVID-19 across China.

**Methods:**

We obtained daily confirmed cases of COVID-19, air particulate matter (PM_2.5_, PM_10_), weather parameters such as ambient temperature (AT) and absolute humidity (AH), and population mobility scale index (MSI) in 63 cities of China on a daily basis (excluding Wuhan) from January 01 to March 02, 2020. Then, the Generalized additive models (GAM) with a quasi-Poisson distribution were fitted to estimate the effects of PM_10_, PM_2.5_ and MSI on daily confirmed COVID-19 cases.

**Results:**

We found each 1 unit increase in daily MSI was significantly positively associated with daily confirmed cases of COVID-19 in all lag days and the strongest estimated RR (1.21, 95% CIs:1.14 ~ 1.28) was observed at lag 014. In PM analysis, we found each 10 μg/m^3^ increase in the concentration of PM_10_ and PM_2.5_ was positively associated with the confirmed cases of COVID-19, and the estimated strongest RRs (both at lag 7) were 1.05 (95% CIs: 1.04, 1.07) and 1.06 (95% CIs: 1.04, 1.07), respectively. A similar trend was also found in all cumulative lag periods (from lag 01 to lag 014). The strongest effects for both PM_10_ and PM_2.5_ were at lag 014, and the RRs of each 10 μg/m^3^ increase were 1.18 (95% CIs:1.14, 1.22) and 1.23 (95% CIs:1.18, 1.29), respectively.

**Conclusions:**

Population mobility and airborne particulate matter may be associated with an increased risk of COVID-19 transmission.

## Background

Coronavirus disease 2019 (COVID-19) was confirmed as a global pandemic by the World Health Organization on March 11, 2020 [1]. COVID-19 is a highly infectious and deadly disease with an estimated basic reproductive number (R_0_) ranging from 2.20 to 3.58 [2–5]. As of October 04, 2020, there have been more than 34.80 million confirmed cases and over 1.03 million deaths worldwide [6]. The world is confronted with an extremely serious public health challenge. China adopted a series of strict prevention and control measures, which include but are not limited to restrictions on crowd gatherings, delays in the start of a new term and return to work, traffic restrictions, health quarantine, free testing services, and treatments. These measures effectively delayed the growth and reduced the size of the COVID-19 epidemic in China [7, 8].

COVID-19 was first confirmed in winter, as the weather with low temperature, mild diurnal temperature range, and low humidity likely contributes to the transmission [9]. Dry and cold environment favors SARS-CoV-2 to survive and transmit in droplets or in the form of aerosols [10]. Previous studies showed that aerosol particles emitted from coughing by influenza patients contain high levels of influenza virus and are within the respirable size range [11]. These virus-accumulating aerosols are easy to transmit among individuals. It was reported that aerosols from highly virulent pathogens like severe acute respiratory syndrome-coronavirus (SARS-CoV) could travel more than six feet [12]. Also, these aerosols are likely composed of airborne pollution particles and attached virus droplets, which promote the spread of pathogens such as influenza viruses [13]. An ecologic analysis found that there were positive relationships between PM_2.5_ concentrations and influenza-like illness risk in Beijing [14]. A cohort study indicated that particulate air pollution was significantly associated with respiratory infections [15]. In particular, the concentrations of airborne ambient particulate matter (PM) with aerodynamic diameter ≤ 2.5 μm (PM_2.5_) were reported to be significantly associated with daily human influenza cases [16, 17], and respiratory syncytial virus infection [18, 19]. In addition to influenza, the SARS outbreak in 2003 was also found to be related to air pollution, as the levels of PM with aerodynamic diameter ≤ 10 μm (PM_10_) were positively associated with the SARS mortality [20]. Like SARS-CoV and influenza viruses, SARS-CoV-2 was detectable in aerosols for up to 3 h, including in both liquid and solid aerosols [21]. A recent study reported that SARS-CoV-2 RNA can be present on outdoor PM, and suggested that, in conditions of atmospheric stability and high concentrations of PM, SARS-CoV-2 could create clusters with outdoor PM and – by reducing their diffusion coefficient – enhance the persistence of the virus in the atmosphere [22]. Setti et al. has emphasized the airborne route as a possible factor for interpreting the anomalous COVID-19 outbreaks in northern Italy, which is characterized by high PM concentrations [23]. Therefore, COVID-19 transmission is likely affected by airborne PM.

Previous studies had found that the population mobility can affect the transmission of infectious diseases, such as the spread of the severe acute respiratory syndrome (SARS) in 2003 [24], the outbreak of the influenza A (H1N1) in 2009 [25], and the transmission of the recurrent human immunodeficiency virus (HIV) [26]. More importantly, the outbreak of COVID-19 in China occurred during the “Spring Festival travel rush”, in which large-scale population mobility may have contributed to the outbreak. As of currently, few studies have found that the size of Wuhan migrants was highly correlated with the daily COVID-19 confirmed cases [27, 28]. One limitation of these studies was that they failed to consider the risk of exposure between population mobility from other areas and Wuhan migrants.

In this study, we established the quasi-Poisson GAM to examine the associations between airborne PM pollution (including PM_10_ and PM_2.5_), MSI and the daily COVID-19 confirmed cases of 63 cities in China while controlling the meteorological factors and other potential factors.

## Methods

### Data collection

Using R package “nCov2019” [29], we obtained the daily COVID-19 confirmed cases of 63 cities in China, each of which confirmed more than 50 cases from January 01 to March 02, 2020. The data of ambient airborne PM, including PM_10_ and PM_2.5_, were obtained from the Data Center of the Ministry of Ecology and Environment of the People’s Republic of China (http://datacenter.mee.gov.cn/). At the same time, the data of ambient temperature (AT) and relative humidity (RH) were collected from the Shanghai Meteorological Bureau. The mobility scale index (MSI) reflects the scale of the population mobility in a city, which can be compared horizontally among cities. We collected the daily MSI for each city from January 01 to March 02. All of the population mobility data were collected from Baidu Migration Map (https://qianxi.baidu.com/). The data from the Baidu Migration Map has been used in previous studies [9, 25], which was considered accurate in estimate the population mobility. Additionally, absolute humidity (AH) was controlled in the models and was calculated via the methods reported previously [30, 31].

### Statistical analysis

We fitted generalized additive models (GAM) with a quasi-Poisson distribution to estimate the associations between airborne PM pollution, MSI, and the daily counts of confirmed cases in each city by controlling the daily average AT, AH, and other potential factors. Due to the overdispersion in COVID-19 transmission [32], we chose quasi-Poisson models to allow for overdispersion in the COVID-19 outbreak among city-days. The models were fitted based on R software (version 3.6.0) with the “mgcv” package (version 1.8–31). The model framework is as follows:

1 $$ \mathrm{Log}\ \mathrm{E}\ \left({\mathrm{Y}}_{\mathrm{t}\mathrm{j}}\right)\kern0.5em =\kern0.5em \upalpha \kern0.5em +\kern0.5em {\upbeta}_1\mathrm{MSI}+\mathrm{ns}\left(\mathrm{AT},\kern0.5em df\right)\kern0.5em +\kern0.5em \mathrm{ns}\left(\mathrm{AH},\kern0.5em df\right)\kern0.5em +\kern0.5em {\upbeta}_2\log \left({\mathrm{Y}}_{\mathrm{t}\hbox{-} 1}\right)+{\mathrm{city}}_{\mathrm{j}} $$

2 $$ \mathrm{Log}\kern0.5em \mathrm{E}\kern0.5em \left({\mathrm{Y}}_{\mathrm{t}\mathrm{j}}\right)=\upalpha +{\upbeta}_1\mathrm{PM}\kern0.5em +\kern0.5em \mathrm{ns}\left(\mathrm{AT},\kern0.5em df\right)+\kern0.5em \mathrm{ns}\left(\mathrm{AH},\kern0.5em df\right)+{\upbeta}_2\mathrm{MSI}\kern0.5em +\kern0.5em {\upbeta}_3\log \left({\mathrm{Y}}_{\mathrm{t}\hbox{-} 1}\right)\kern0.5em +\kern0.5em {\mathrm{city}}_{\mathrm{j}} $$

In these model, t refers to the day of the observation; j refers to the cities. Y_tj_ is the observed daily confirmed case counts in city j on day t; E (Y_tj_) is the expected daily confirmed case counts in city j on day t; α is the intercept; β represents the regression coefficient; MSI represents mobility scale index in city j on day t (model 1); airborne PM, including PM_10_ and PM_2.5_, represents concentrations in city j on day t; log (Y_t-1_) indicates the log-transformed COVID-19 counts on day t-1 in city j to control the potential serial autocorrelation [33]. We used a natural smooth function (ns) with 6 *df* for 3-day moving average AT and 3 *df* for 3-day moving average AH to control potential nonlinear and lagged confounding effects of weather conditions. Considering the collinearity and latent period of COVID-19, 7-day moving average MSI was controlled in the models (2) when exploring the effects of PM_10_ and PM_2.5_. The city_j_ indicates city fixed effects to control for city-specific characteristics such as population density [34].

Because the latent period of COVID-19 ranges from 1 to 14 days, mostly 3 to 7 days [35], we chose to estimate the single-day lag effects (from lag 1 to lag 14) and cumulative lag effects (from lag 01 to lag 014). The results were expressed as the relative risk (RR) and 95% confidence intervals (CIs) for each 10 μg/m^3^ increase in PM_2.5_ and PM_10_ concentrations or each 1 unit increase in MSI. In order to intuitively observe the impact of airborne PM and MSI on the daily COVID-19 confirmed cases, we plotted the exposure-response curves based on GAM model (1) and model (2) to analyze the relationship between changes of the PM_2.5_, PM_10_, and MSI in lag 07 and lag 014 days and the daily COVID-19 confirmed cases.

Due to the stricter control measures implemented in Hubei compared with those in other cities, we conducted a subgroup analysis to present the effects in the cities from Hubei province and the cities outside Hubei separately. Previous studies used the GAM with a Gaussian distribution to assess the association of air pollutants and human mobility with daily COVID-19 confirmed cases [36, 37], so we conducted a sensitivity analysis by applied the GAM with a Gaussian distribution to assess the association of airborne PM and MSI with daily COVID-19 confirmed cases. Besides, we firstly used the GAM with a quasi-Poisson distribution to estimate city-specific effects of PM_10_, PM_2.5_, and MSI on daily confirmed COVID-19 cases. Then, the random effects model of meta-analysis was used to pool the city-specific effects (Supplementary Material Methods S1).

All statistical tests were two-sided, and *p*-values less than 0.05 were considered as statistically significant.

## Results

**Fig. 1:**
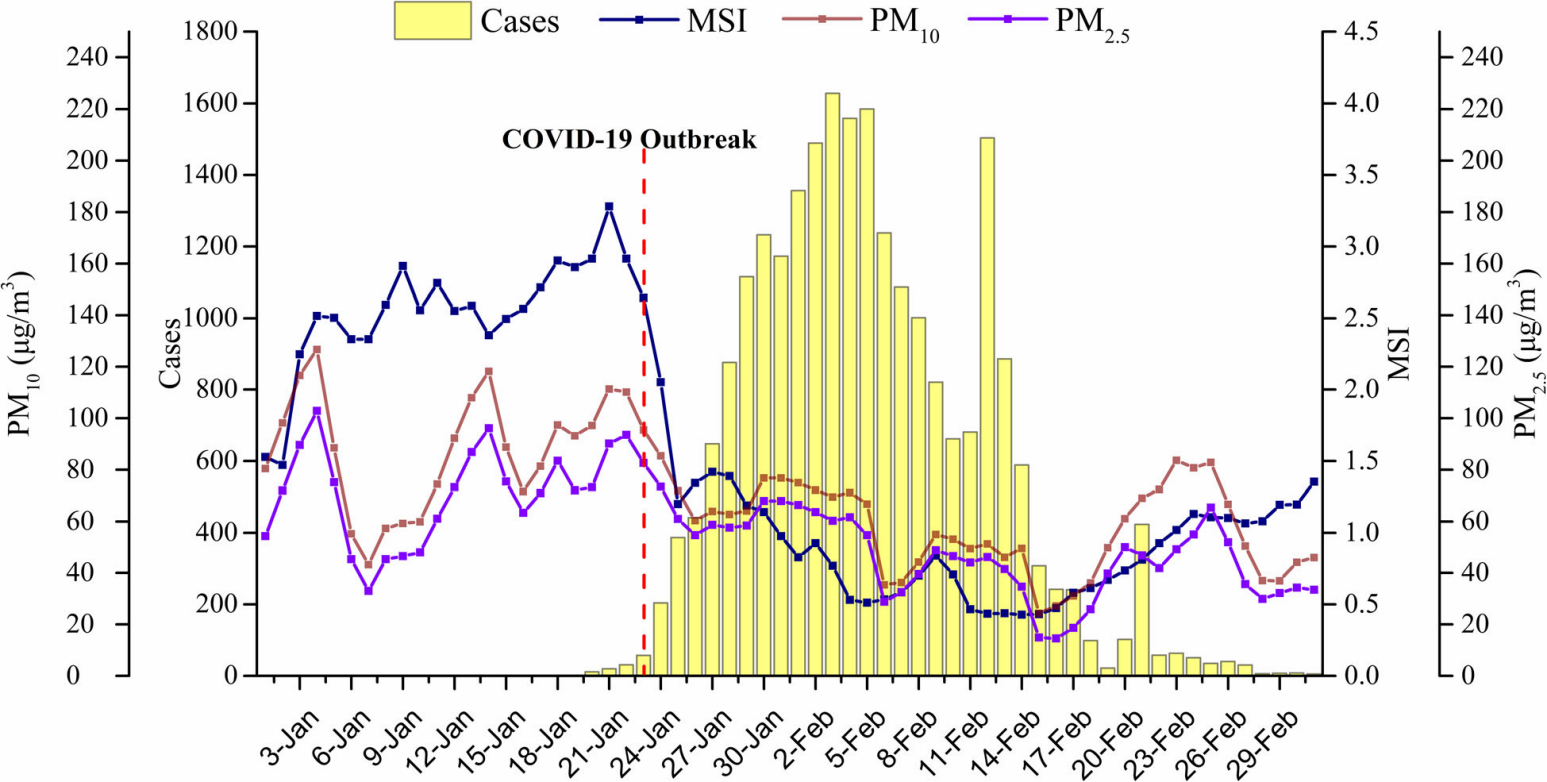
Trends of daily PM levels, MSI, and confirmed COVID-19 cases in 63 cities of China from January 01 to March 02, 2020

In these 63 cities, 37 cities confirmed more than 100 cases (22,229, 92.56%) and 26 cities confirmed less than 100 cases (1787, 7.44%), among which the 12 Hubei cities (excluding Wuhan) confirmed the most cases (16,759, 69.78%). During the disease reporting period, the average AT and AH ranges in each city were − 0.75 ~ 16.52 °C and 2.75 ~ 11.28 g/m^3^, respectively. The outbreak of COVID 19 in 63 cities began on January 23, peaked on February 03, then began to decline, and gradually approached 0 after February 28 (Fig. 1). The daily average concentration of PM_10_ and PM_2.5_ exhibited a similar trend, in which both declined significantly after January 23 (Fig. 1). From January 01 to January 23, the MSI rose slowly with the Spring Festival approaching, declined rapidly and stabilized after January 23, but then began to rise slowly after February 15 (Fig. 1). From January 24 to March 02, the MSI of 63 cities decreased by an average of 64.78% per day compared with those from January 01 to January 23.

**Fig. 2:**
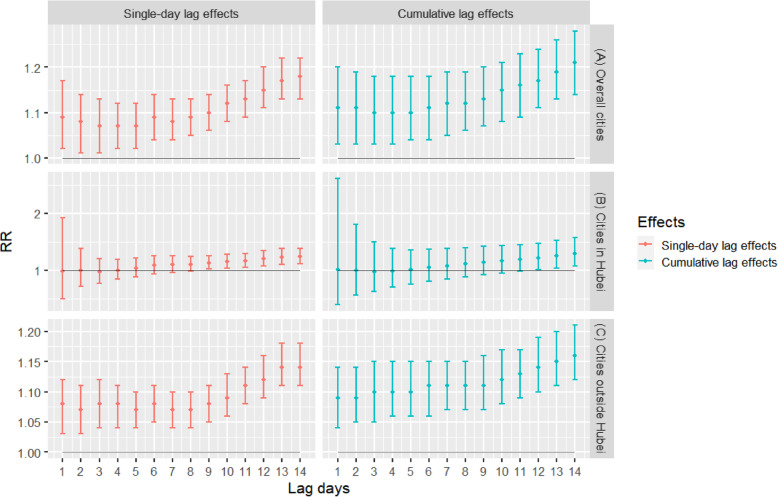
Associations between MSI and the COVID-19 confirmed cases in 63 cities of China from January 01 to March 02, 2020. Note: The results were expressed as the relative risk (RR) and 95% confidence intervals (CIs) for each 1 unit increase in MSI

**Fig. 3:**
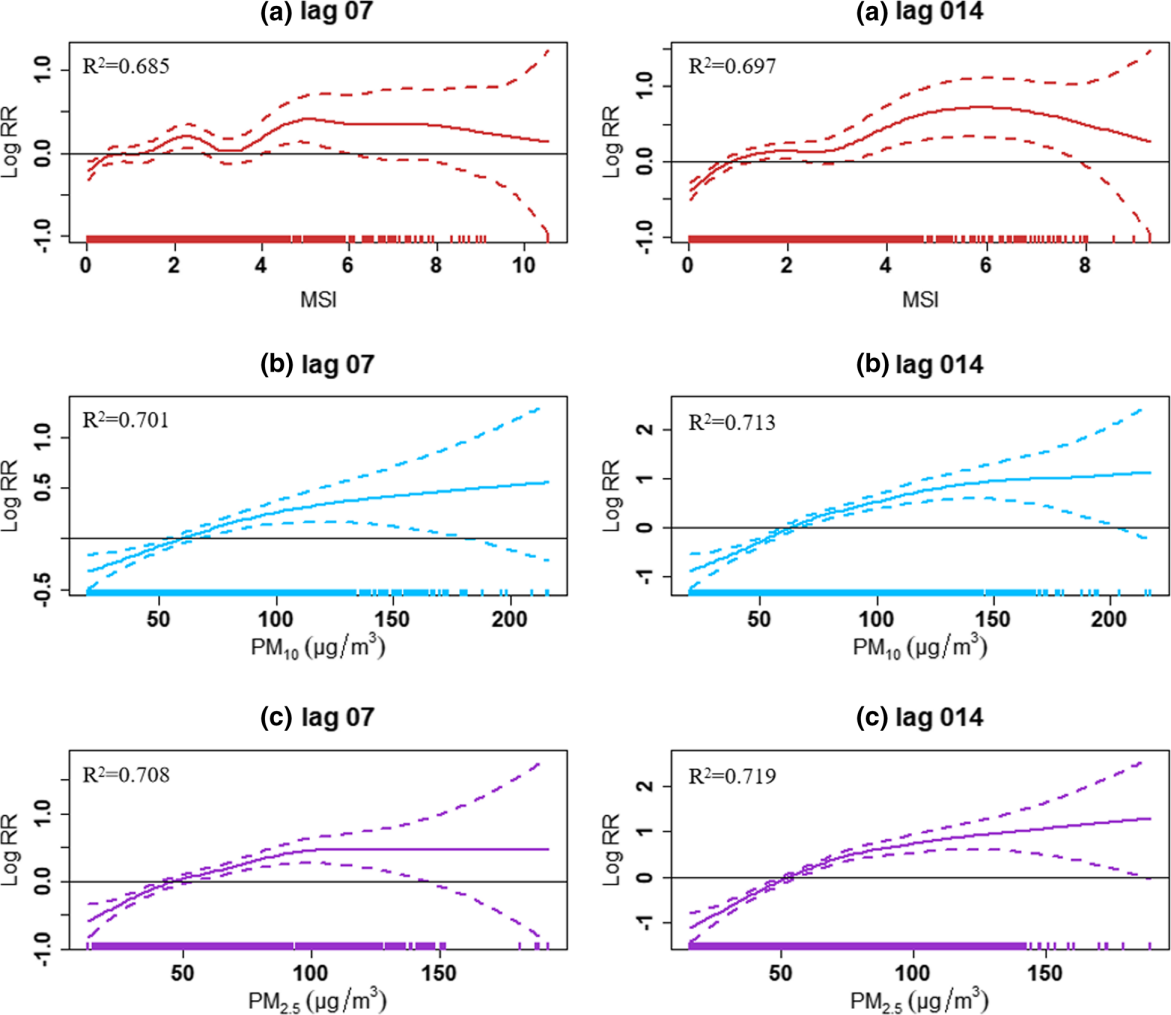
The exposure-response curves of MSI, PM_10_, PM_2.5_ and the daily COVID-19 confirmed cases in 63 cities of China from January 01 to March 02, 2020. Note: (**a**) MSI; (**b**) PM_10_; (**c**) PM_2.5_. The X-axis is the values of MSI, PM_10_, PM_2.5_ in lag 07 or lag 014 days, Y-axis is the predicted log relative risk (RR), is shown by th color solid line, and the color dotted lines represent the 95% confidence interval (CI). The R^2^ represents the fitting effect, and the closer R^2^ is to 1, the better the fitting effect of the model

As shown in Fig. 2 and Fig. 3a, there are significant positive associations between the daily confirmed COVID-19 cases and MSI. We found each 1 unit increase in the daily MSI was positively and significantly associated with the daily confirmed cases of COVID-19 in 63 cities at all lag days, while the estimated strongest RR at lag 14 was 1.18 (95% CIs:1.13, 1.22). For cumulative lag effects, the estimates of 63 cities were statistically significant in all lag days, while the strongest RR at lag 014 was 1.21 (95% CIs:1.14, 1.28) (Fig. 2a). For the cities in Hubei, the strongest single-day effects for MSI were at lag 14, while the RR of each 1 unit increase was 1.24 (95% CIs:1.11, 1.39). The cumulative lag effects were the strongest for MSI at lag 014, and the corresponding RR was 1.29 (95% CIs:1.07, 1.57) (Fig. 2b). For the cities outside Hubei, the strongest effects of single-day effects for MSI were at lag 14, and the RR was 1.14 (95% CIs:1.11, 1.18). The strongest effects of cumulative lag effects for MSI were at lag 014, and the RR was 1.16 (95% CIs:1.12, 1.21) (Fig. 2c).

**Fig. 4:**
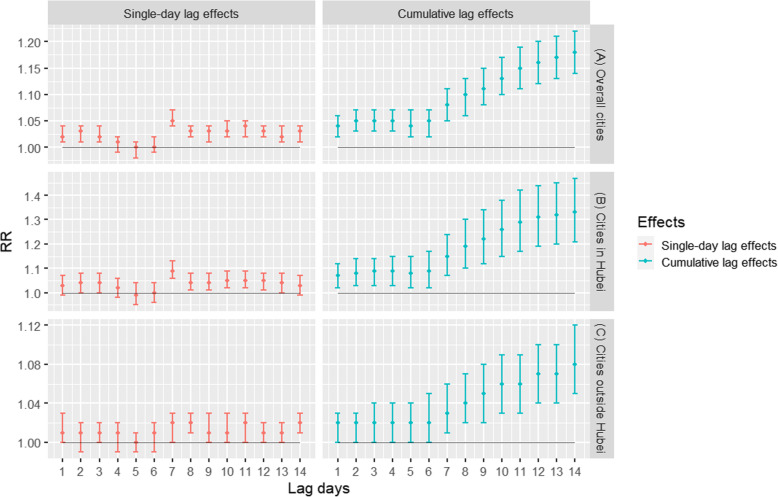
Associations between PM_10_ and the COVID-19 confirmed cases in 63 cities of China from January 01 to March 02, 2020. Note: The results were expressed as the relative risk (RR) and 95% confidence intervals (CIs) for each 10 μg/m^3^ increase in PM_10_ concentrations

**Fig. 5:**
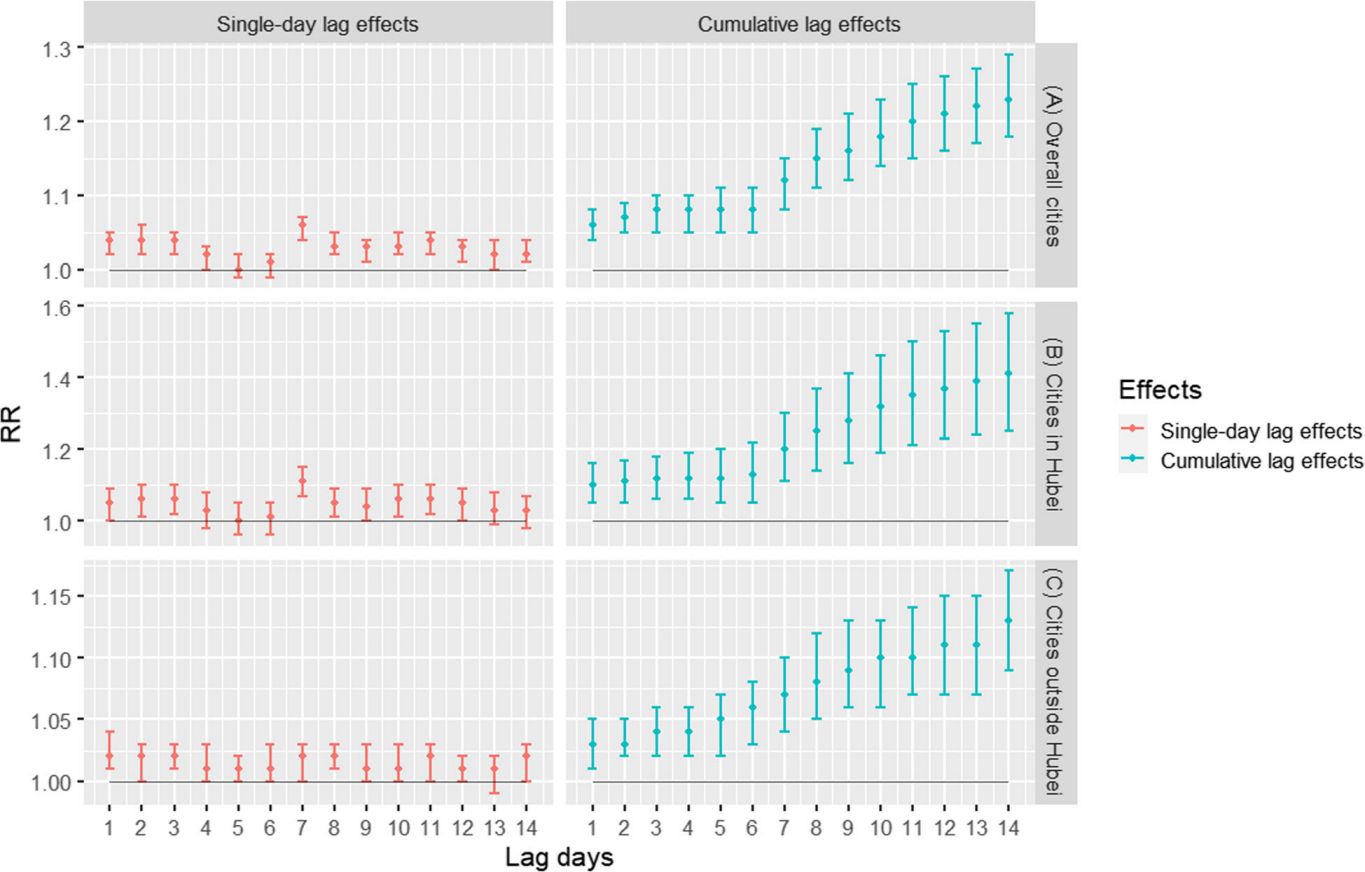
Associations between PM_2.5_ and the COVID-19 confirmed cases in 63 cities of China from January 01to March 02, 2020. Note: The results were expressed as the relative risk (RR) and 95% confidence intervals (CIs) for each 10 μg/m^3^ increase in PM_2.5_ concentrations

As shown in Fig. 4a, Fig. 5a, and Fig. 3b and c, there were significant positive associations between the daily confirmed COVID-19 cases and particulate matter pollution in 63 cities. For PM_10_ and PM_2.5_, the strongest single-day effects were at lag 7, and the corresponding RRs were 1.05 (95% CIs: 1.04, 1.07) and 1.06 (95% CIs: 1.04, 1.07), respectively. For cumulative lag effects, the estimates of 63 cities were all significant with the strongest effects for both PM_10_ and PM_2.5_ appearing in lag 014, while the RRs of each 10 μg/m^3^ increase were 1.18 (95% CIs:1.14, 1.22) and 1.23 (95% CIs:1.18, 1.29), respectively. Additionally, the overall effects were much higher for PM_2.5_ than PM_10_.

Figures 4b and c and Fig. 5b and c showed the associations between airborne particulate matter pollution and the confirmed cases stratified by the cities from Hubei province and the cities outside Hubei, respectively. For the cities in Hubei, the strongest single-day effects for PM_10_ and PM_2.5_ were both at lag 7, and the RRs of each 10 μg/m^3^ increase were 1.09 (95% CIs:1.06, 1.13) and 1.11 (95% CIs:1.07, 1.15), respectively. The strongest cumulative lag effects for PM_10_ and PM_2.5_ were both at lag 014, and the corresponding RRs of which were 1.33 (95% CIs:1.21, 1.47) and 1.41 (95% CIs: 1.25, 1.58), respectively. For the cities outside Hubei, the strongest effects of single-day effects for both PM_10_ and PM_2.5_ were at lag 8, and the RRs were 1.02 (95% CIs:1.01, 1.03) and 1.02 (95% CIs:1.01, 1.03). The strongest cumulative lag effects for both PM_10_ and PM_2.5_ appeared in lag 014, and the RRs of each 10 μg/m^3^ increase were 1.08 (95% CIs:1.05, 1.12) and 1.13 (95% CIs:1.09, 1.27), respectively. Furthermore, stronger associations between COVID-19 and airborne PM were found for cities inside Hubei than outside Hubei

The effects estimated by the GAM with a Gaussian distribution are similar to the GAM with a quasi-Poisson distribution, but the GAM with a quasi-Poisson distribution has a better fitting effect (Supplementary Material Fig.S1 and Table S1). The pooled effects from the city-specific result are the same as the model (1) and model (2). The MSI, PM_10_ and PM_2.5_ at lag 014 have a greater effect on the daily confirmed COVID-19 case. But the model fitting effect of the city-specific is poor (Supplementary Material Fig. S2 and Table S2).

## Discussion

Aerosols have been recently confirmed as a potential transmission route for SARS-CoV-2 [38], which may be modified by the level of airborne PM pollution. Besides, population mobility was also one important contributor toward the transmission of COVID-19. In controlling the population mobility, meteorological factors as well as other potential factors, we found positive relationships between PM_2.5_, PM_10_, and daily confirmed COVID-19 cases counts in China.

As infectious diseases, population mobility might lead to wide transmissions among different regions. Our current study has found that population mobility was positively related to the daily confirmed COVID-19 case counts. Meanwhile, because of the apparent influence of meteorological factors in COVID-19 transmission [9, 39], we controlled meteorological factors and MSI in our models to clarify the associations between COVID-19 case counts and airborne PM pollution. After controlling these factors, we found both PM_2.5_ and PM_10_ were positively related to the COVID-19 confirmed cases, suggesting that airborne PM pollution might affect COVID-19 transmission. This was similar to other studies focusing on influenza and SARS. Specifically, a study conducted in 47 Chinese cities has found that ambient PM_2.5_ concentrations may increase the risk of exposure to influenza in China, especially during days with low temperatures [40]. Croft et al. have found that short-term increases in traffic and other combustion source-related PM_2.5_ might contribute to the increased rates of influenza hospitalizations [41]. Jaspers et al. reported that diesel exhaust increased influenza virus attached to respiratory epithelial cells within 2 h post-infection [42]. Furthermore, long-range transportation of influenza virus A was found during dust storm days with higher concentrations of ambient influenza A virus [43]. By conducting an ecologic study, Cui et al. found that the case fatality rate of SARS in 5 regions increased with the increment of air pollution index (API); in particular, those patients in regions with higher API suffered greater risks of death [44]. In addition, Liu et al. have reported the presence of SARS-CoV-2 on airborne particles inside Wuhan Hospitals and in the surroundings by on-field studies [38]. And Santarpia et al. have also reported the presence of SARS-CoV-2 on air samples collected at the University of Nebraska Medical Center [45]. Similar findings are reported in Bergamo of Northern Italy, where SARS-CoV-2 RNA was found on air particulate matter [22]. Concerning particles’ role in the viral diffusion process, there is a hypothesis that aerosol droplets emitted by infected persons during sneezing, coughing or simply talking are stabilized in the air through the coalescence with PM at high concentrations and under conditions of atmospheric stability [46]. Therefore, higher levels of airborne particulate matter may increase the transmission of COVID-19.

Airborne PM pollution is a health hazard that could be deposited deep in the lungs and impair immune function [47, 48]. Research studies reported that airborne PM decreased the ability of pulmonary macrophages to effectively mount a defense against infection, which would last at least a week post-exposure via RelB activation [49]. Since pulmonary macrophages are very important in lung to phagocytize pathogens, the suppression of that function would increase the invasive ability of SARS-CoV-2. Also, airborne PM induces respiratory inflammation and affects the health of the airway [50, 51]. In particular, these severe inflammation in the lung after exposure to PM_2.5_ were found to be mediated by angiotensin-converting enzyme 2 (ACE2), which showed a significant increase in the lung after PM_2.5_ exposure [52]. Interestingly, it is reported that the receptor-binding domain of the SARS-CoV-2 could be recognized by the extracellular peptidase domain of ACE2, which is predominantly expressed in a transient secretory cell type in subsegmental bronchial branches [53, 54]. Thus, the airborne PM may increase the possibility of SARS-CoV-2 lung invasion through the ACE2 pathway. Altogether, these evidence may explain the reason for the positive association between airborne PM and COVID-19.

According to epidemiological investigations, the latent period of COVID-19 is 1 ~ 14 days, most of which is 3 ~ 7 days [35]. Therefore, the newly infected arrivals and the second-generation infected persons were diagnosed as COVID-19 cases with a time lag. Our results showed that the MSI in single lag 14 days and cumulative lag 14 days had the greatest effect on the daily confirmed COVID-19 cases. This shows that the time from the infected people replicated second-generation infected objects to the second-generation infected objects were diagnosed as COVID-19 cases are mostly 14 days. The PM_10_ and PM_2.5_ in single lag 7 days and cumulative lag 14 days had the greatest effect on the daily confirmed COVID-19 case in cities in Hubei. But the strongest effect of single-day effects for both PM_10_ and PM_2.5_ were at lag 8 day in the cities outside Hubei. This indicates that most people in cities in Hubei were confirmed on the 7th day after infecting SARS-CoV-2, while cities outside Hubei were confirmed on the 8th day after infecting SARS-CoV-2. This is related to the influence of testing methods, testing ability and reporting procedures and other factors. Generally, the cumulative lag effect is greater than the single-day lag effect, and the effect increases with the prolonging of the cumulative lag time. Zhu et al. have also shown that air pollutants and population mobility index at a long cumulative lag period have a greater effect on the daily confirmed COVID-19 case [36, 37]. The outbreak of COVID-19 in China occurred during the “Spring Festival travel rush, in which large-scale population mobility may have contributed to the outbreak. According to Yang et al. model, the government’s administrative actions effectively reduced the size of the spread of COVID-19 [7], which was mainly related to the decline in population mobility. The measures of limiting population mobility effectively delayed the arrival time of the epidemic peak [8], and allowed for a sufficient amount of time to respond to the outbreak for other provinces and cities.

### Limitations

There are some potential limitations in this study. First, some other factors may affect the incidence of COVID-19, such as public health interventions, but we examined the impact of air pollution after controlling the population mobility and meteorological factors. Second, there were modifications of COVID-19 case definitions at different stages of the epidemic, which may affect the confirmed counts. To reduce the bias as a result of the altering definition, we included 63 cities with more than 50 confirmed cases in our analysis. In addition, since the diagnosis of COVID-19 cases is largely influenced by governmental screening standards, especially in Wuhan, we unequivocally decided to exclude Wuhan in this study. Finally, the study was only conducted in China although the COVID-19 is recognized as an emergent world pandemic. Therefore, our conclusions require future evaluation with global data. Despite these limitations, our study provided some evidence from multiple cities across China and increased the scope of knowledge in elucidating the effect of PM pollution and population mobility on COVID-19. Further investigations that include more globally detailed data on public health interventions and individual-level characteristics would be critical to study the associations between air pollution, population mobility, and COVID-19.

## Conclusion

Our findings indicate that population mobility and airborne particulate matter may be associated with an increased risk of COVID-19 transmission. Thus, population mobility need to be controlled in fighting against COVID-19 epidemic. We suggest that it is necessary to pay attention to the potential effect of PM on COVID-19 transmission. However, ecological fallacy and many uncontrolled confounding factors such as different public health interventions may have biased our results. Future studies are needed to real-time test the presence of SARS-CoV-2 adsorbed on air PM and assess its vitality and virulence in COVID-19 epidemic areas.

## Supplementary information

**Additional file 1.**

## Data Availability

The daily COVID-19 confirmed cases were obtained from R package “nCov2019” [29], which is publicly available and does not contain any individual persons information. The population mobility datasets and particulate matter datasets used and/or analyzed during the current study are available from the open-access websites. The meteorological datasets used and/or analyzed during the current study are not publicly available but are available from the corresponding author on reasonable request.

## Abbreviations

COVID-19: Coronavirus disease 2019
PM: Particulate matter
PM_2.5_: Particulate matter with aerodynamic diameter ≤ 2.5 μm
PM_10_: Particulate matter with aerodynamic diameter ≤ 10 μm
MSI: People mobility scale index
AT: Ambient temperature
RH: Relative humidity
AH: Absolute humidity
GAM: Generalized additive models
RR: Relative risk
CIs: Confidence intervals
