# Supplementary material for: Airborne particulate matter, population mobility and COVID-19: a multi-city study in China
Source: BMC Public Health. 2020 Oct 21;20:1585. doi: 10.1186/s12889-020-09669-3 (PMC7576551; doi:10.1186/s12889-020-09669-3)
Supplement: Supplementary file 1 — Additional file 1. [file 12889_2020_9669_MOESM1_ESM.docx]

**Supplementary Material**

**Methods S1. The two-stage analysis to estimate the effects of airborne PM and population mobility on COVID-19 cases.**

In the first stage, the Generalized additive models (GAM) with quasi-Poisson distribution were firstly fitted to estimate city-specific effects of PM_10_, PM_2.5_, and MSI on daily confirmed COVID-19 cases while controlling ambient temperature (AT) and absolute humidity (AH). The GAM model framework is as follows :

Log E (Y_t_) = α + β_1_MSI + ns(AT, *df* ) + ns(AH, *df* ) + β_2_log(Y_t-1_) (3)

Log E (Y_t_) = α + β_1_PM + ns(AT, *df* ) + ns(AH, *df* ) + β_2_MSI + β_3_log(Y_t-1_) (4)

In these model, t refers to the day of the observation; Y_t_ is the observed daily confirmed case counts on day t; E(Y_t_) is the expected daily confirmed case counts on day t; MSI represents MSI on day t (model 3); airborne PM, including PM_10_ and PM_2.5_, represents concentrations on day t; log(Y_t-1_) indicates the log-transformed COVID-19 counts at day t-1 to control the potential serial autocorrelation. We used a natural smooth function (ns) with 6 *df* for 3-day moving average AT and 3 *df* for 3-day moving average AH to control potential nonlinear and lagged confounding effects of weather conditions. Considering the collinearity and latent period of COVID-19, 7-day moving average MSI was controlled in the models (4) when exploring the effects of PM_10_ and PM_2.5_.

In the second stage, the random effects model of meta-analysis was used to pool the city-specific effects of the PM10, PM2.5, and MSI. The meta-analysis was based on R software “meta” package (version 4.11-0).


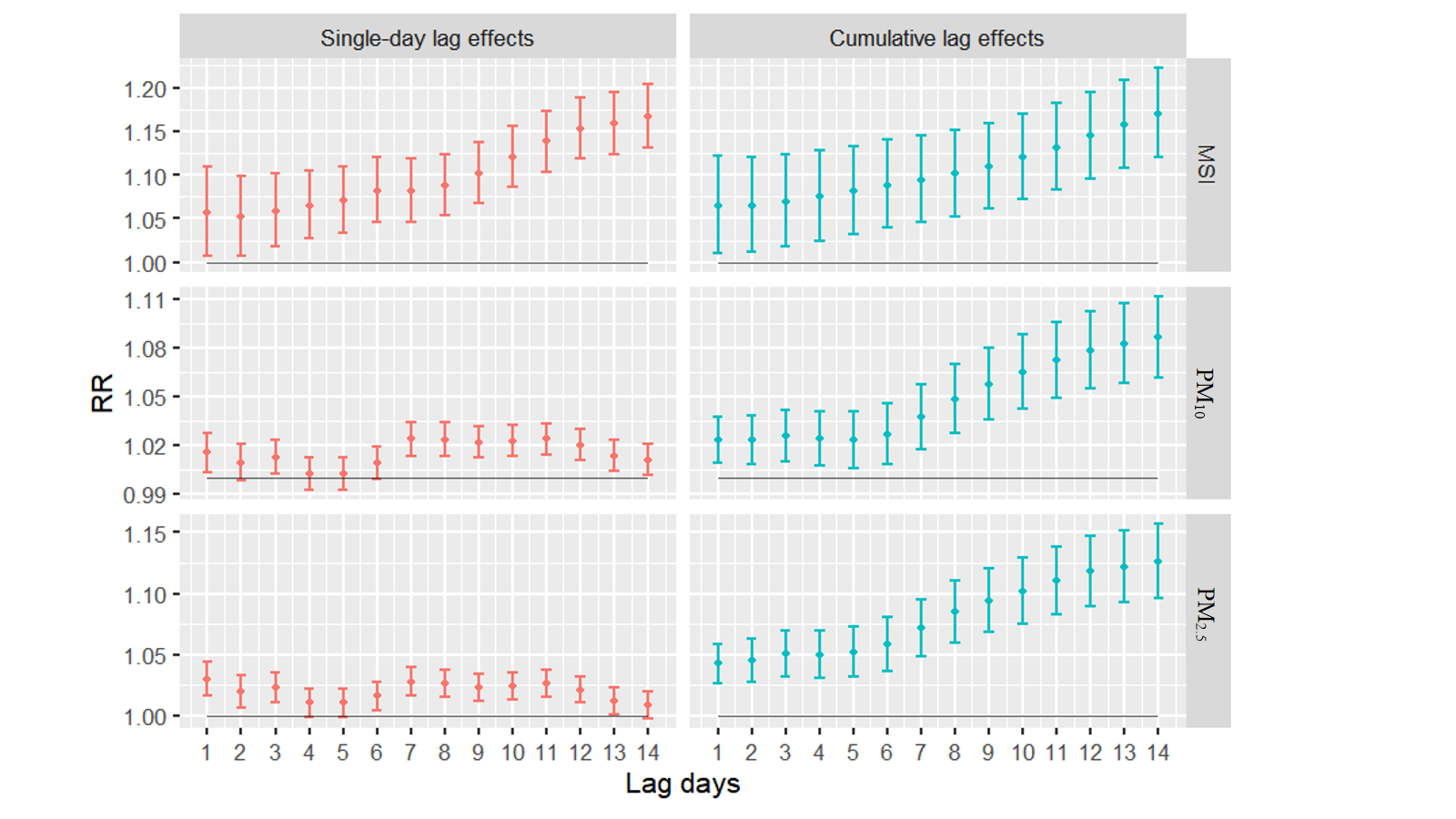
**Fig. S1. Associations between MSI, PM_10_, PM_2.5_ and the daily COVID-19 confirmed cases in 63 cities of China from January 20 to March 02, 2020, based on model (5) and model (6).**

Note: The effects based on the Generalized additive model (GAM) with a Gaussian distribution. The results were expressed as the relative risk (RR) and 95% confidence intervals (CIs) for each 1 unit increase in MSI or 10 μg/m^3^ increase in PM_10_ and PM_2.5_ concentrations. The models was defined as follows:

log (Y_tj_) = α + β_1_MSI + ns(AT, *df* ) + ns(AH, *df* ) + β_2_log(Y_t-1_) +city_j_ (5)

log (Y_tj_) = α + β_1_PM + ns(AT, *df* ) + ns(AH, *df* ) + β_2_MSI + β_3_log(Y_t-1_) + city_j_ (6)

Here, the log(Y_tj_) indicates the logarithm of the number of confirmed new cases in city j on day t (plus 1 to avoid taking the logarithm of 0). Other covariates is the same as model (1) and model (2).

**Table S1**. **Summary of model fitting results.**

| **Lag days** | **R^2^ (MSI)** | | **R^2^ (PM_10_ )** | | **R^2^ (PM_2.5_)** | |
| --- | --- | --- | --- | --- | --- | --- |
|  | Model (1)  quasi-Poisson  distribution | Model (5)  Gaussian  distribution | Model (2)  quasi-Poisson  distribution | Model (6)  Gaussian  distribution | Model (2)  quasi-Poisson  distribution | Model (6)  Gaussian  distribution |
| lag 1 | 0.679 | 0.648 | 0.685 | 0.653 | 0.687 | 0.653 |
| lag 2 | 0.679 | 0.648 | 0.689 | 0.652 | 0.692 | 0.651 |
| lag 3 | 0.679 | 0.648 | 0.688 | 0.653 | 0.692 | 0.652 |
| lag 4 | 0.679 | 0.649 | 0.682 | 0.652 | 0.683 | 0.650 |
| lag 5 | 0.679 | 0.650 | 0.680 | 0.652 | 0.679 | 0.650 |
| lag 6 | 0.680 | 0.651 | 0.680 | 0.652 | 0.678 | 0.651 |
| lag 7 | 0.679 | 0.651 | 0.693 | 0.656 | 0.693 | 0.654 |
| lag 8 | 0.679 | 0.652 | 0.679 | 0.656 | 0.677 | 0.653 |
| lag 9 | 0.681 | 0.654 | 0.680 | 0.655 | 0.679 | 0.652 |
| lag 10 | 0.681 | 0.657 | 0.684 | 0.656 | 0.683 | 0.653 |
| lag 11 | 0.683 | 0.660 | 0.680 | 0.656 | 0.680 | 0.653 |
| lag 12 | 0.691 | 0.663 | 0.682 | 0.655 | 0.680 | 0.652 |
| lag 13 | 0.700 | 0.664 | 0.686 | 0.653 | 0.683 | 0.650 |
| lag 14 | 0.699 | 0.665 | 0.685 | 0.653 | 0.683 | 0.650 |
| lag 01 | 0.679 | 0.648 | 0.696 | 0.654 | 0.701 | 0.655 |
| lag 02 | 0.679 | 0.648 | 0.697 | 0.653 | 0.703 | 0.654 |
| lag 03 | 0.679 | 0.648 | 0.698 | 0.654 | 0.705 | 0.655 |
| lag 04 | 0.679 | 0.648 | 0.696 | 0.653 | 0.704 | 0.654 |
| lag 05 | 0.679 | 0.649 | 0.692 | 0.653 | 0.699 | 0.654 |
| lag 06 | 0.679 | 0.649 | 0.690 | 0.653 | 0.696 | 0.655 |
| lag 07 | 0.679 | 0.650 | 0.696 | 0.654 | 0.703 | 0.657 |
| lag 08 | 0.679 | 0.650 | 0.699 | 0.656 | 0.706 | 0.658 |
| lag 09 | 0.680 | 0.651 | 0.700 | 0.657 | 0.707 | 0.660 |
| lag 010 | 0.680 | 0.652 | 0.703 | 0.658 | 0.710 | 0.661 |
| lag 011 | 0.681 | 0.653 | 0.705 | 0.659 | 0.711 | 0.662 |
| lag 012 | 0.682 | 0.654 | 0.705 | 0.660 | 0.711 | 0.663 |
| lag 013 | 0.684 | 0.655 | 0.708 | 0.661 | 0.713 | 0.663 |
| lag 014 | 0.686 | 0.657 | 0.709 | 0.661 | 0.714 | 0.663 |

|  |
| --- |

Note: The R^2^ represents the fitting effect of the model, and the closer R^2^ is to 1, the better the fitting effect of the model.


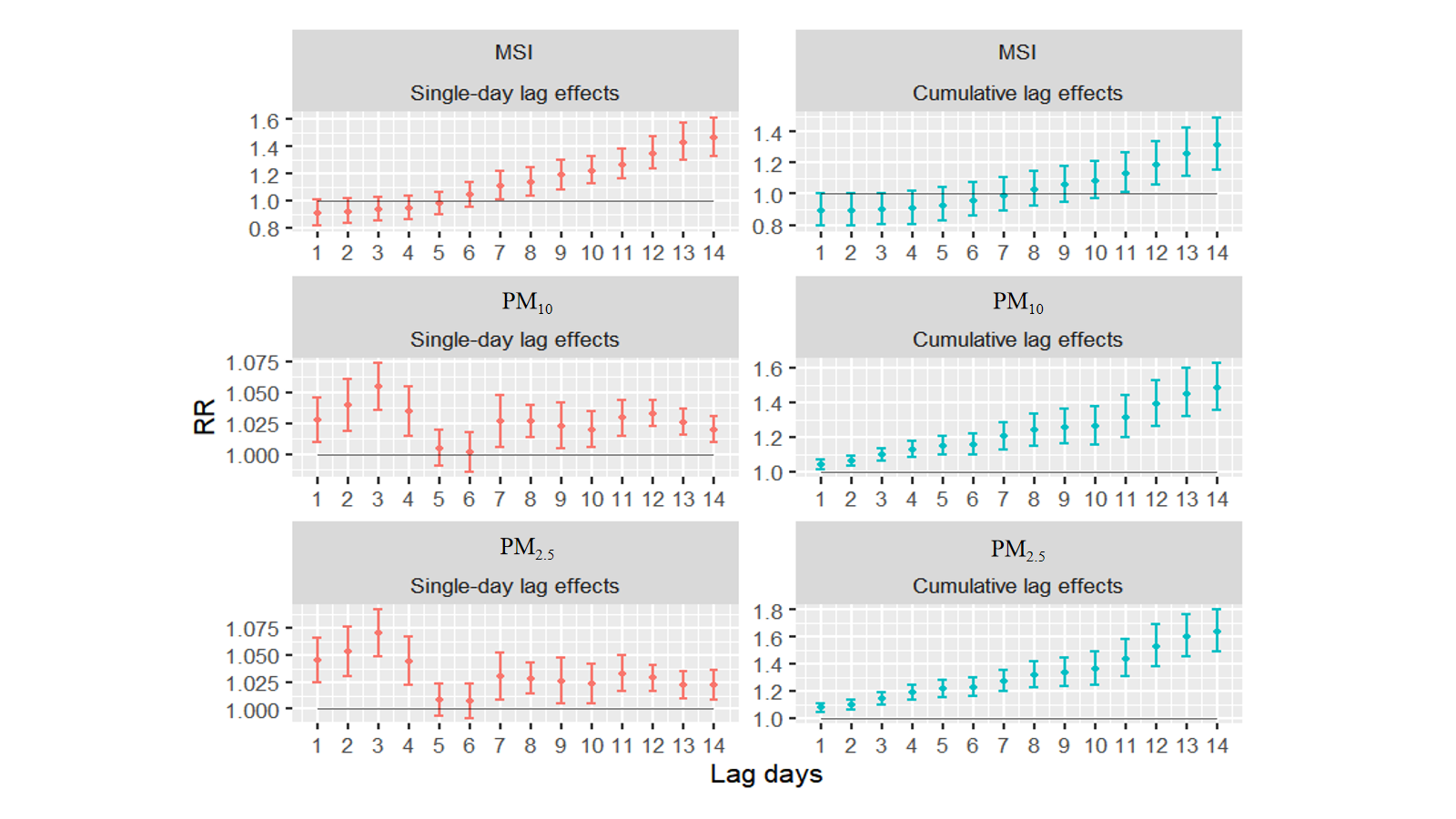
**Fig. S2.** **Associations between MSI, PM_10_, PM_2.5_ and the daily COVID-19 confirmed cases in 63 cities of China from January 20 to March 02, 2020, based on model (3) and model (4).**

Note: The RR is the pooled effect of 63 cities. The results were expressed as the relative risk (RR) and 95% confidence intervals (CIs) for each 1 unit increase in MSI or 10 μg/m^3^ increase in PM_10_ and PM_2.5_ concentrations.

**Table S2**. **Summary of model fitting results in 10 cities.**

| **City** | **PM_10_** | | **PM_2.5_** | | **MSI** | |
| --- | --- | --- | --- | --- | --- | --- |
|  | R^2^ (lag 7) | R^2^ (lag 014) | R^2^ (lag 7) | R^2^ (lag 014) | R^2^ (lag 7) | R^2^ (lag 014) |
| Beijing | 0.659 | 0.725 | 0.633 | 0.736 | 0.611 | 0.653 |
| Qinqdao | 0.653 | 0.328 | 0.325 | 0.325 | 0.282 | 0.303 |
| Tangshan | 0.706 | 0.769 | 0.722 | 0.679 | 0.599 | 0.648 |
| Suizhou | 0.411 | 0.749 | 0.419 | 0.806 | 0.422 | 0.438 |
| Yiyang | 0.391 | 0.192 | 0.366 | 0.192 | 0.151 | 0.171 |
| Nanjing | 0.421 | 0.401 | 0.423 | 0.389 | 0.457 | 0.429 |
| Shanghai | 0.761 | 0.721 | 0.801 | 0.766 | 0.741 | 0.744 |
| Kunmimg | 0.612 | 0.715 | 0.611 | 0.741 | 0.627 | 0.623 |
| Changde | 0.536 | 0.503 | 0.533 | 0.537 | 0.439 | 0.369 |
| Jining | 0.505 | 0.471 | 0.517 | 0.469 | 0.494 | 0.398 |

Note: Due to the large number of cities in the study, the model fitting results of only 10 cities are shown here. The R^2^ represents the fitting effect of the model, and the closer R^2^ is to 1, the better the fitting effect of the model.
